# Occlusal features and need for orthodontic treatment in persons with osteogenesis imperfecta

**DOI:** 10.1002/cre2.53

**Published:** 2017-02-09

**Authors:** Minh Son Nguyen, Ho Duy Binh, Khac Minh Nguyen, Katre Maasalu, Sulev Kõks, Aare Märtson, Mare Saag, Triin Jagomägi

**Affiliations:** ^1^ Institute of Dentistry University of Tartu Tartu Estonia; ^2^ Danang University of Medical Technology and Pharmacy Danang Vietnam; ^3^ Hue University of Medicine and Pharmacy Hue Vietnam; ^4^ Clinic of Traumatology and Orthopaedics University of Tartu Tartu Estonia; ^5^ Department of Pathophysiology University of Tartu Tartu Estonia

**Keywords:** Index of Orthodontic Treatment Need, malocclusion, osteogenesis imperfecta

## Abstract

The aim of the study was to (a) analyse dental occlusion and determine the need for orthodontic treatment of persons with osteogenesis imperfecta (OI) in comparison with the healthy population and (b) investigate the associations between OI and malocclusion. A case‐control study included 26 OI persons and 400 healthy participants (control group). Occlusal features and the need for orthodontic treatment were defined according to Dental Health Component‐Index of Orthodontic Treatment Need and Dental Aesthetic Index. Results showed that Angle Class I, II, and III relationship was found in 23.1%, 3.8%, and 73.1% of OI group, and in the control group, it was 67%, 17.5%, and 15.5%, respectively. OI group had significantly higher prevalence of reverse overjet >1 mm (76.9%), missing teeth (42.3%), posterior crossbite (34.6%), and open bite >2 mm (19.2%) compared to the control group (8.5%, 2.2%, 6.2%, and 3.5%, respectively). OI group had less incisal segment crowding and more incisal segment spacing than the control group (*p* < 0.05). The need for orthodontic treatment of OI group according to Dental Health Component‐Index of Orthodontic Treatment Need and Dental Aesthetic Index was 88.5% and 61.5%, respectively, while in the control group, it was 24.8% and 51.8%. The malocclusion in OI persons was associated with reverse overjet > 1 mm (OR = 13.3, 95% CI = 3.9–44.7, *p* < .001), Angle Class III malocclusion (OR = 8.0, 95% CI = 2.0–30.8, *p* = .003), and missing teeth (OR = 4.7, 95% CI = 1.0–22.4, *p* = .049). In conclusion, there is the high probability of malocclusion in OI persons. Persons with OI require early orthodontic treatment because of significant correlation of OI disease with Angle Class III malocclusion, reverse overjet, and missing teeth.

## 1 INTRODUCTION

Osteogenesis imperfecta (OI), also known as brittle bone disease, is a congenital bone disorder related to type 1 collagen alpha chains (COL1A1 and COL1A2) metabolism disorder that affects skeletal development. The incidence of OI is estimated to be one per 20,000 live births (Andersen & Hauge, 1989; Rauch & Glorieux, 2004). According to the Sillence's classification, OI is divided into four types, based on clinical features and disease severity (Sillence, Senn, & Danks, 1979). Type I is the mildest form of the disorder. Type II, the most severe case, is often lethal in the perinatal period. Type III is the most serious form of surviving patients. Type IV is of intermediate clinical features between Type I and III. Recently, the classification has been more expanded into several types related to genetic findings (Rauch & Glorieux, 2004).

OI primarily affects patients' skeletal structures resulting in decreased bone mineral density, increased fracture risk, skeletal deformations, hypermobile joints, and short stature. The disease also influences similar type I collagen‐rich structures and causing the symptoms of hearing loss and discoloration of the sclera (Lin et al., 2009; Sillence et al., 1979).

Dentinogenesis imperfecta has been associated with OI, regarding dental–facial manifestations (Lin et al., 2009; Opsahl Vital et al., 2012; Saeves, Axelsson, Wekre, & Storhaug, 2006). Other oral problems of persons with OI are also reported in the literature. Persons with OI often have poor oral health and disharmony of maxilla and mandible that is considered high risk for development of malocclusion (Chang, Lin, & Hsu, 2007; Saeves et al., 2009; Stenvik, Larheim, & Storhaug, 1985). Clinical observations have shown occlusion of OI persons seem to be related to Angle Class III malocclusion, anterior and posterior crossbites, open bite, and missing teeth (Rizkallah et al., 2013; Saeves et al., 2006; Saeves et al., 2009). The data concerning the comparison of occlusion between OI and the healthy population and defining the risk of malocclusion are however still insufficient. Moreover, data on the orthodontic treatment need for persons with OI have not yet been reported in the literature.

Brook and Shaw introduced the Dental Health Component‐Index of Orthodontic Treatment Need (DHC‐IOTN; Brook & Shaw, 1989). The DHC‐IOTN classifies malocclusions according to the presence of particular occlusal features, considered necessary to identify individuals who would benefit most from the orthodontic treatment. Dental Aesthetic Index (DAI) is recommended by World Health Organization to establish the orthodontic treatment need, based on total score of assessment of ten occlusal features (Cons, Jenny, Kohout, Songpaisan, & Jotikastira, 1989). Both DHC‐IOTN and DAI are widely applied in epidemiologic studies.

The aim of the study was to (a) analyse dental occlusion and determine the need for orthodontic treatment of persons with OI in comparison with the healthy population based on using the DHC‐IOTN and DAI and (b) investigate the associations between OI and malocclusion.

## 2 MATERIALS AND METHODS

### 2.1 Osteogenesis imperfecta sample

Data were collected from OI persons in five medical centres in Vietnam. One hundred fourteen OI persons agreed to participate; among them, 62 persons were under 11 years old, 26 were aged from 12 to 16 years, and the others were in adult age group. In this study, we selected the group of 12–16 years old (n = 26) because permanent dentition of this age group could establish a stable occlusal relationship and eliminate the bias of the visible missing teeth variable; therefore, their occlusions were in accordance with criteria of DHC‐IOTN and DAI.

Diagnosis of OI was confirmed based on clinical and radiological characteristics by two orthopaedic surgery specialists. The distribution of types of OI according to Sillence's classification included Type I (n = 7), Type III (n = 10), and Type IV (n = 9); however, we only focus on analyses of malocclusion of OI persons in this study. The associations between each type of OI and dental occlusion will be investigated in further research. Most of OI persons had not yet received bisphosphonate therapy.

### 2.2 Control group

The control group consisted of 400 participants including 200 12‐year‐old school children and 200 18‐year‐old students. School children were randomly selected from five primary schools, and students were from among the 4,000 students studying at the Danang University of Medical Technology and Pharmacy in Danang City, Vietnam.

### 2.3 Control bias

All subjects had no history of orthodontic treatment, and the mean of age between OI and control groups was statistical equivalence (OI group = 16.8, control group = 15.0, *p* ≥ .05).

### 2.4 Recording dental occlusion

The DHC‐IOTN defined particular occlusal features including overjet >3.5 mm, overbite >3.5 mm, open bite >2 mm, contact point displacement >2 mm, reverse overjet >1 mm, posterior crossbite, and missing teeth. All these parameters were used to determine if the subject had a borderline need for orthodontic treatment.

To determine the DAI, 10 occlusal features were evaluated including number of visible missing maxillary teeth (including anterior teeth and premolars), number of visible missing mandibular teeth (including anterior teeth and premolars), anterior maxillary overjet (mm), anterior mandibular overjet (mm), midline diastema (mm), vertical anterior open bite (mm), the largest anterior maxillary irregularity (mm), the largest anterior mandibular irregularity (mm), incisal segment crowding (segment scores 0–2), and incisal segment spacing (segment scores 0–2). The Angle's molar relationship was also recorded.

Dental examination of OI group was performed at medical centres while the control group was examined in the dental clinic of the Danang University of Medical Technology and Pharmacy. Intraoral radiography was not performed for participants during the dental examination.

A pilot study of a group of 20 participants of the control group was performed to calibrate the examiner. The dental examination of both groups was carried out by the first author. The OI persons were reexamined in the same day and 10% of the control group was reexamined after 3 days to test the reliability of the examiner. The calculated Kappa values were above 0.85, indicating a high degree of intra‐examiner and inter‐examination reliability.

### 2.5 Determination of the need for orthodontic treatment

The DHC‐IOTN has five grades used to evaluate the need for treatment based on the worst occlusal feature of participant: Grades 1 and 2 equally suggest no or little treatment needed; Grade 3 represents a borderline need for treatment; and Grades 4 and 5 indicate a high priority for treatment.

The DAI evaluates the need for orthodontic treatment, based on total score of ten occlusal features: DAI score ≤ 25 represents no or a slight need for treatment; DAI score 26–30 indicates elective treatment; DAI score 31–35 equals treatment highly desirable, and DAI score ≥ 36 represents treatment definitely needed.

DHC‐IOTN grades and DAI scores for the degree of treatment needed were reclassified into two groups: “without treatment needs” (DAI < 31; DHC‐IOTN Grades 1–3) and “in need of treatment” (DAI ≥ 31; DHC‐IOTN Grades 4–5).

Written informed consent that explained oral examination procedures was obtained from each participant or their parents. The study was approved by the ethical review board of Hue University Hospital (No. 75/CN‐BVYD), the Danang University of Medical Technology and Pharmacy (No 523/CN‐DHKTYDDN), and the University of Tartu (No. 221/M‐34). All procedures were performed according to the World Medical Association Declaration of Helsinki.

### 2.6 Statistical analysis

The data were analysed using with Version 17.0 of the Statistical Package for the Social Sciences software (SPSS Inc., Chicago, Illinois, USA). Chi‐square and Student's *t* test determined the significance difference of occlusal features and the need for orthodontic treatment of DHC‐IOTN and DAI between OI and control groups. Binomial logistic regression estimated the odds of having malocclusion among OI group compared to control group. The confidence level at 95% and a two‐sided *p* value of .05 were used for the significant difference.

## 3 RESULTS

Of 26 OI persons, 30.8% were females and 69.2% were males. Of 400 selected participants in the control group, females and males were 58.5% and 41.5%, respectively. According to the Angle's classification, the prevalence of Class I, Class II, and Class III malocclusion of the OI group was 23.1%, 3.8%, and 73.1% compared to the control group with 67%, 17.5%, and 15.5%, respectively (Table 1).

**Table 1:** Occlusal features based on Dental Health Component‐Index of Orthodontic Treatment Need (DHC‐IOTN) and Dental Aesthetic Index (DAI) of osteogenesis imperfecta (OI) persons compared to the control group

Occlusal feature	OI group	Control group	*p* value
	n = 26	n = 400	
Angle's molar relationship(%)^a^			
Class I	23.1	67.0	<.001*
Class II	3.8	17.5	.090
Class III	73.1	15.5	<.001*
DHC‐IOTN (%)^a^			
Overjet > 3.5 mm	0.0	36.2	<.001*
Overbite > 3.5 mm	26.9	26.0	.917
Open bite > 2 mm	19.2	3.5	.004*
Contact point displacement > 2 mm	46.2	54.0	.437
Reverse overjet > 1 mm	76.9	8.5	<.001*
Posterior crossbite	34.6	6.2	<.001*
Missing teeth	42.3	2.2	<.001*
DAI^b^			
Number of missing visible maxillary teeth^c^	0.58	0.02	.003*
Number of missing visible mandibular teeth^c^	0.31	0.03	.030*
Anterior maxillary overjet (mm)	1.44	3.13	.004*
Anterior mandibular overjet (mm)	3.94	2.67	.026*
Midline diastema (mm)	0.46	0.16	.225
Vertical anterior open bite (mm)	0.54	0.14	.122
Largest anterior maxillary irregularity(mm)	1.04	1.42	.325
Largest anterior mandibular irregularity (mm)	0.89	1.43	<.001*
Incisal segment crowding^d^	0.54	1.00	.005*
Incisal segment spacing^d^	0.58	0.25	.040*

* Significant.

a Chi‐square test;

b Student's *t* test;

c Including incisors, canines, and premolars.

d Incisal segment scores 0–2.

Regarding DHC‐IOTN, there were significant differences between OI and control groups, respectively, in terms of overjet > 3.5 mm (0.0% and 36.2%, *p* < .001), open bite >2 mm (19.2% and 3.5%, *p* = .004), reverse overjet >1 mm (76.9% and 8.5%, *p* < .001), posterior crossbite (34.6% and 6.2%, *p* < .001), and missing teeth (42.3% and 2.2%, *p* < .001, Table 1)

According to DAI, most of the occlusal features were found to differ significantly between OI and control groups, except for midline diastema, vertical anterior open bite, and the largest anterior maxillary irregularity. OI group had a lower score of anterior maxillary overjet (1.44 mm) but a higher score of anterior mandibular overjet (3.94 mm) than the control group (3.13 mm and 2.67 mm, respectively, *p* < .05). The mean number of missing teeth, including anterior teeth and premolars in the OI group, was also much higher than in the control group (*p* < .05). The mean number of segments with incisal crowding of OI group (0.54) was less than in the control group (1.0, *p* = .005), but those of incisal spacing of OI group (0.58) was higher than in the control group (0.25, *p* = .040). The mean score of the largest mandibular anterior teeth irregularity of the control group (1.43 mm) was statistically higher than that of the OI group (0.89 mm, *p* < .001, Table 1).

Approximately 89% of OI persons with DHC‐IOTN and 61.5% with DAI definitely need orthodontic treatment, which was a statistically significant difference compared with the control group (24.8% and 51.8%, respectively, *p* < .001, Table 2).

**Table 2:** Prevalence of the need for orthodontic treatment of osteogenesis imperfecta (OI) and control groups

The need for orthodontic treatment	OI group	Control group	*p* value
DHC‐IOTN grade			
Grades 1–3 (No need treatment)	11.5	75.2	<.001*
Grades 4–5 (Definitive treatment)	88.5	24.8	
DAI score			
Score < 31 (No need treatment)	38.5	48.2	<.001*
Score ≥ 31 (Definitive treatment)	61.5	51.8	

* Significant.

a *Note*. DHC‐IOTN = Dental Health Component‐Index of Orthodontic Treatment Need; DAI = Dental Aesthetic Index.

b Chi‐square test;

Table 3 shows that OI was positively associated with reverse overjet >1 mm (OR = 13.3, 95% CI = 3.9–44.7, *p* < .001), Angle Class III malocclusion (OR = 8.0, 95% CI = 2.0–30.8, *p* = .003), and missing teeth (OR = 4.7, 95% CI = 1.0–22.4, *p* = .049) but negatively associated with contact point displacement >2 mm (OR = 0.1, 95% CI = 0.02–0.42, *p* = .002). The need for orthodontic treatment of OI group with DHC‐IOTN Grades 4–5 was 27.3 times much more than in the control group (*p* < .001).

**Table 3:** The odds of malocclusion of osteogenesis imperfecta group compared to the control group

Variable	OR	95% CI	*p* value
Contact point displacement > 2 mm			
No	1.0 (ref.)		
Yes	0.1	0.02–0.42	.002*
Missing teeth			
No	1.0 (ref.)		
Yes	4.7	1.0–22.4	.049*
Angle Class III malocclusion			
No	1.0 (ref.)		
Yes	8.0	2.0–30.8	.003*
Reverse overjet > 1 mm			
No	1.0 (ref.)		
Yes	13.3	3.9–44.7	<.001*
DHC‐IOTN grade			
Grades 1–3	1.0 (ref.)		
Grades 4–5	27.3	5.1–144.8	<.001*

* Significant.

a *Note*. OR = odds ratio; CI = confidence interval; DHC‐IOTN = Dental Health Component‐Index of Orthodontic Treatment Need.

b Binomial logistic regression;

c ref: reference.

## 4 DISCUSSION

OI is a genetic disorder resulting in increased bone fragility and low‐bone mass (Rauch & Glorieux, 2004). The incidence of person with OI is estimated at 1:20,000 of the population; therefore, only the small number of OI patients related to oral findings was reported in previous studies; for example, 49 OI sufferers (5–19 years old) among 100 patients were selected for research conducted in Canada (Rizkallah et al., 2013); 16 OI patients in Taiwan (Chang et al., 2007); 94 OI patients with age over 25 years old in Norway (Saeves et al., 2009); Lin et al. collected 48 OI patients for 8 years in his research in Taiwan (Lin et al., 2009). The sample of our study consisted of 114 OI persons; however, we only selected 26 OI persons ranging from age 12–16 years for our study. The explanation was that selected participants had permanent dentition and stable occlusal relationship, eliminating the bias of the visible missing teeth variable of DHC‐IOTN and DAI.

In general, Angle's classification of occlusion has been used in epidemiological studies to evaluate occlusal relationships. We found that the distribution of Class I (23.1%), Class II (3.8%), and Class III (73.1%) malocclusions of the OI group was significantly biased compared to the control group (67%, 17.5%, and 15.5%, respectively). The high proportion of Angle Class III malocclusion for our OI persons is in line with the research of Rizkallah (57%) (Rizkallah et al., 2013) and Chang (62.5%) (Chang et al., 2007).

Angle Class III malocclusion presented the obvious difference between both groups in the current study. In addition, although the DHC‐IOTN reflected qualitative occlusal features and the DAI measured quantitative occlusal parameters, both indexes indicated the main problems of OI group were related to severe reverse overjet. This finding is in accordance with the characteristic features of the Class III pattern. For this reason, we also hypothesized that collagen mutation in the foetal phase might cause structural disorders of collagen in the mandibular and condylar regions, so it leads to the severe Class III malocclusion and reverse overjet.

Open bite is the concerning issue for OI persons. In the current study, the prevalence of OI persons with open bite >2 mm was significantly higher than the control group. This finding could be explained by the fact that the gonial angle of the mandible of OI persons was larger compared to those of the non‐OI persons. Moreover, spinal curvature and triangular face of OI persons might result in the mandible resting on their chest and thereby also result in an anterior rotation of the mandible and open bite (Chang et al., 2007; Rizkallah et al., 2013).

Our finding is consistent with the study of Rikallah et al. (Rizkallah et al., 2013) showing that midline diastema was not a significant difference between OI patients and healthy population seeking for orthodontic treatment. Rikallah's study also concluded that the OI syndrome did not affect facial symmetry.

Nonetheless, the study of Chang et al. demonstrated the poor growth of the maxilla based on analysing cephalometric radiographs of OI patients (Chang et al., 2007). This finding could explain for our results that no OI person had overjet > 3.5 mm, which was often observed in the control group (36.2%). The transverse and sagittal discrepancies of the maxilla of OI persons were evidenced related to more posterior crossbite and less anterior maxillary overjet as compared to the control group. Similar results were obtained in a study conducted on the patients suffering from OI in Canada (Rizkallah et al., 2013).

The presence of maxillary deficiencies among OI persons might cause tooth crowding owing to a lack of (Rizkallah et al., 2013; Schindel & Duffy, 2007), but interestingly, the prevalence of contact point displacement > 2 mm and the score of largest anterior maxillary irregularity of OI group were relatively similar compared to the control group who had normal facial skeletons. By using the discrepancy index, another instrument to measure malocclusion, the previous study also confirmed that dental crowding in OI patients was not different from the healthy people seeking for orthodontic treatment (Rizkallah et al., 2013). Even though, dental crowding might not be considered as prominent malocclusion for OI group in this study. The evidence presented that OI group had more incisal segment spacing and less incisal segment crowding than the control group. In many cases, missing of the teeth probably compensated for the lack of interdental space in OI persons.

As we predicted, the prevalence and number of missing teeth of OI group was significantly higher than those of the control group. The prevalence of missing teeth in general populations might be up to 11% according to the epidemiological study (Shivakumar, Chandu, Subba Reddy, & Shafiulla, 2009). We revealed that 42.3% of individuals, affected by OI, had missing teeth, whereas only 2.2% of the control group. The cause of missing teeth could be tooth extraction, delayed tooth eruption, hypodontia, or abnormal odontogenesis; however, it has been still unclear for OI persons of our study and in previous research (Saeves et al., 2009).

In the current study, missing teeth of OI group could be referred for genetic disorders that would cause hypodontia. Genetic disorders disease such as cleft lip and palate, ectodermal dysplasia, or Down syndrome are important causes of hypodontia (AlShahrani, Togoo, & AlQarni, 2013; Cobourne, 2007). Similar studies also list mutation of collagen type III alpha 1 (COL3A1) in the hypermobility type of Ehlers–Danlos syndrome as associated with hypodontia (Chhabra, Goswami, & Chhabra, 2014; Giunta et al., 2008). The candidate genes, therefore, causing collagen mutation in OI persons might likewise be responsible for the hypodontia that was found in this study.

The binary logistic regression also revealed that missing teeth were significantly more often in OI group (OR = 4.7) in the current study. The odds ratio of our study was much higher compared to the finding published by Saeves et al. in 2009 (OR = 2.0) (Saeves et al., 2009). The difference might be that the age group of OI patients of Saeves' study was over 25 years old from European origin while participants in our sample were Vietnamese children and adolescents aged from 12–16 years.

Although the DHC‐IOTN and DAI evaluated occlusal features in the different ways, both indexes described in detail the severity of malocclusions and determined the need for orthodontic treatment for OI and control groups. In this study, 88.5% OI persons with the DHC‐IOTN Grades 4–5 and 61.5% with DAI score ≥ 31 definitely need orthodontic treatment; this was much higher compared to the control group (OR = 27.3). The need for orthodontic treatment of the control group was only in the range of 24.8–51.8%, depending on the used index.

We found that there was a slight difference in the determination of the need for orthodontic treatment between using the DHC‐IOTN and DAI. The most significant finding from our study was that in OI persons with simultaneous occurrence of missing teeth and reverse overjet, it is enough to identify the need for orthodontic treatment based on the DHC‐IOTN, whereas the DAI is more complicated, being dependent on the score of 10 occlusal features.

Our study indicated that OI persons had the odds of having malocclusion associated with reverse overjet > 1 mm, Angle Class III malocclusion, and missing teeth based on clinical examination; however, the limitation of our study is that we could not use lateral cephalogram and orthopantomography to analyse facial skeletal pattern and missing teeth because medical centres specialized in care for OI persons had not been equipped with those radiographic devices yet. Furthermore, although the number of persons with different types of OI was mentioned in the methods of the study, the associations between types of OI and malocclusion did not present in the results, because our sample is relatively small with only 26 children and adolescents. A larger sample should be necessary to analyse risks of malocclusion for each type of OI.

## 5 CONCLUSION

There was a high probability of reverse overjet, missing teeth, and Angle Class III malocclusion in OI persons. Both DHC‐IOTN and DAI indicated that large proportion of persons with OI definitely needs orthodontic treatment. As OI is a disease that causes physical disability and decreases the quality of life, the OI persons need medical and dental care to maintain their social activity.

## CONFLICT OF INTEREST

No conflict of interest.
